# Predictors of delayed and no-reflow as recognized with Thrombolysis in Myocardial Infarction [TIMI] flow grade following Primary Percutaneous Coronary Angioplasty

**Published:** 2015

**Authors:** M Bahrehmand, E Sadeghi, A Shafiee, Y Nozari

**Affiliations:** * Department of Cardiology, Kermanshah University of Medical Sciences, Kermanshah, Iran,; ** Department of Surgery, Kermanshah University of Medical Sciences, Kermanshah, Iran,; *** Tehran Heart Center, Tehran University of Medical Sciences, Tehran, Iran

**Keywords:** TIMI Flow, myocardial infarction, no reflow, subcutaneous Coronary interposition

## Abstract

**Background:** Initial percutaneous coronary interference (PCI) is still connected by a noticeable incidence of suboptimal coronary flow thrombolysis in infarction of myocardial (TIMI). The predictors of slow and no-reflow in cases that supported initial PCI in our institute was searched for and the relationship of these parameters with major adverse cardiovascular effects (MACE) was assessed.

**Material and Method:** 397 patients with AMI displaying in 24 hours of the sign opening were retrospectively enrolled and underwent primary PCI between March 2006 and March 2012. Demographic, clinical, and procedural data were retrieved from our institutional databank. The baseline and post-PCI flow of blood in the revascularized artery was ranked based on the TIMI grading method. The follow-up visits were performed after one, six and twelve month from hospitalization. All the mortalities and complications were recorded within this period for evaluate the MACE.

**Results:** The frequency of diabetes mellitus and renal failure were importantly larger in cases with a TIMI flow of 0-1 (p=0.03 & p=.01, respectively). Similarly, level of serum creatine were importantly larger in cases with a TIMI flow of 0-1. The predictors for TIMI flow included that utilize of Adenosin or Integrilin, diabetes mellitus, POIT, long tubular lesion, and injury at LAD territory. The incidence of MACE was significantly higher in patients with a TIMI flow of 0-1 (P=0.001) and the survival in this subgroup was significantly poorer (Hazard ratio=4.96; P<0.001).

**Conclusion:** A low TIMI flow is accompanied by a poorer survival and a higher MACE and is influenced by some clinical and vascular characteristics.

## Introduction

Primary subcutaneous Coronary interposition (PCI) has become the therapy of choice for serious infarction of myocardial (AMI) (AMI). In spite of the advances in the stenting and angioplasty procedures, initial PCI was still associated with 4–11% incidence of flow of suboptimal coronary thrombolysis in myocardial infarction (TIMI) [**1**-**4**]. Current evidence shows that the AMI patients with angiographic suboptimal reflow have a weak functional recovery and a higher rate of post-AMI complications compared to the patients with an optimal reflow [**3**,**5**,**6**]. While the TIMI tree current following PCI is a significant prophesied of the result in cases by AMI, patients with a TIMI flow of up to grade 2 had a poor prognosis [**7**-**8**].

No-reflow is described as suboptimal myocardial reperfusion in a section of coronary flow out angiographically proving a obstruction of mechanical vessel [**9**]. Several factors, including age, infarct localization, the extent of the primary AMI area, the loss of remaining blood flow into the infarct-related artery, prior AMI, raised C-reactive protein levels can enhance the danger of poor final coronary blood flow [**8**,**10**]. Although several methods have been used to increase the success of PCI and reduce the death and disease [**11**], that identification of the predictors of reperfusion failure can help increase the procedure quality and thereby, its rate of success. 

In the running research, we aimed to find out the predictors of slow and no-reflow in cases that supported initial PCI in our institute. Moreover, we assessed the relationship of these parameters with important adverse cardiovascular effects. 

## Material and Method

In this cohort study, we retrospectively enrolled 397 patients with AMI displaying in 24 hours of the sign start and underwent primary PCI between March 2006 and March 2012 in Tehran Heart Center. Heart Center of Tehran is a 460-bed tertiary center for cardiovascular diseases, affiliated to Medical Sciences Tehran University in Iran. Patients who were treated via thrombolysis or coronary artery bypass grafting (CABG) operation was no involved. Data of the enrolled patients, including the clinical, demographic and procedural parameters, are reclaimed of the angioplasty databank of core [**12**]. 

All the recruited patients had approved a signed data approval at the time of admission, declaring that their clinical data could be used anonymously for research. The study protocol was approved by the Research Board of Heart Center of Tehran, and the Committee of Medical Ethics of Medical Sciences Tehran University.

The ST-segment elevation severe myocardial infarction was diagnosed in this proximity of heartache doing for more than 20 minutes connected with the electrocardiographic differences (S-T-segment rise of more than one mm into limited to end electrocardiographic signs or more than two mm in at limited two adjacent precordial heads or new onset left package part section). The analysis is verified with coronary angiography in every case.

The angiography and PCI is worked in the catheterization Tehran Heart Center lab below regional numbness. All angioplasty procedures are done based on modern standard guidelines [**13**-**15**]. All the patients received 325 mil gram orally Aspirin, 600 mg Clopidogrel, 80 mg statin and a measurement changed intravenous

Heparin bolus (100 IU/ kg) prior to PCI. Stenting is done in higher than 91 percent of the cases, bare metal stents being mostly used. The angiography videos were revised by a cardiologist, who was unaware of the study protocol, to improve the intra- and inter-observer reliability. The baseline and post-PCI flow of blood into the revascularized artery was graded based on TIMI grading system [**16**]. In fact, type zero perfusion expressed not antegrade movement away the occlusion; type two is a minimum, inadequate perfusion of contrast average round the mass; type three (partial perfusion) is a perfect just limited perfusion from the distal coronary bed by contrast element; and type three (complete perfusion) is an antegrade movement to the whole distal artery at a regular flow. The analysis of no-reflow is assigned according to the next tests: [**1**] angiographic proof of opening the occluded coronary artery & the strong stent situation by not proof of flow-limiting remaining stenosis (<50%), spasm, dissection, or apparent thrombus and [**2**] angiographic evidence of a TIMI current type ≤2, at limited ten minutes later the completion of the PCI system.

The post-PCI antiplatelet treatment include clopidogrel (75-milligrams/d to a limited one to six months) & aspirin (80-milligrams/d given orally). The other cardiac conditions were treated according to the judgment of the responsible physician. The follow-up visits were routinely performed after 1, 6, and 12 months from hospitalization in our center. All the mortalities and cardiac related complications are shown in the duration and are utilized to evaluate the MACE. MACE is described via in-hospital death, cardiac mortality, nonfatal myocardial infarction (MI), or purpose artery revascularization. In-hospital MI is diagnosed in the beginning 7 days then the system if further unusual Q waves are recognized by a rise in serum creatine kinase-MB (CK-MB) isoenzyme or only an rise in CK-MB further than threefold in the cause of loss of the Q waves [**17**-**18**].

**Statistical Analysis**

The mean ± standard deviation or median with quartiles, and frequency (percentage) are utilized to describe the continuing and absolute changeable. The continuous variables were compared between the TIMI groups by using the student’s t or Mann-Whitney U test. Categorical variables were compared between the mentioned groups by using chi-square or the Fisher’s exact test. A multivariate logistic regression study is done to define the clinical and angiographic changeable that could independently predict the poor post-interventional coronary reflow. All information are prepared by the PASW, version 18 (USA, Chicago, Illinois). P-values smaller than or equivalent to 0.051 are analyzed statistically notable. 

## Results

The existing research consisted of 397 patients (mean age = 56.57 ± 12.43 years), male gender = 312 (78.6%) whose data were reviewed and who underwent an elective coronary angiography in our center. Slow/ no-reflow occurred in 18 (4.5%) patients. Baseline specifications of the research themes are compared according to the final TIMI flow grade subgroups as explained in **Tab. 1**. Regarding this comparison, the diabetes mellitus frequency and failure of renal were significantly higher in cases by a TIMI flow grade of 0-1 (p=0.03 and p=0.01, respectively). Similarly, serum levels of creatine importantly larger at cases by TIMI flow type = 0-1. The use of aspirin, beta-blockers, nitrates and clopidogrel was importantly larger at cases by a TIMI flow of two (p=0.001, p=0.02, p=0.004, and p=0.004, respectively). Also, utilize of adenosine and integrilin is importantly larger in cases by TIMI flow grade two (p< 0.001 for both). also, the use of lipid lowering agents is higher frequency in cases by a TIMI flow type 0-1. 

**Table 1:** Baseline specifications of the research people

Characteristics	TIMI = 0,1 (n=18)	TIMI = 2 (n=151)	TIMI = 3 (n=228)	P-value*
Age	57.94 ± 16.78	56.69 ± 11.67	56.39 ± 12.58	0.86
Male gender	11 (61.1)	123 (81.5)	178 (78.1)	0.13
BMI	28.62 ± 4.05	27.30 ± 3.76	26.85 ± 4.20	0.31
Abdominal circumference	107.5 (97.0, 113.5)	99.0 (93.5, 105.5)	99.0 (93.0, 106.0)	0.11
Medical history				
Family history of CAD	2 (11.1)	25 (16.6)	49 (21.5)	0.33
Diabetes mellitus	9 (50.0)	50 (33.6)	54 (23.8)	0.01
Hypertension	9 (50.0)	67 (45.0)	102 (44.9)	0.91
Dyslipidemia	10 (55.6)	106 (71.6)	150 (66.7)	0.3
Smoking				
Current	3 (16.7)	49 (32.9)	77 (33.9)	
Former	1 (5.6)	19 (12.8)	20 (8.8)	
Stable angina	0 (0)	10 (6.6)	23 (10.1)	0.2
Unstable angina	1 (5.6)	19 (12.6)	33 (14.5)	0.53
Angina pectoris	9 (50.0)	76 (50.3)	123 (53.9)	0.62
STEMI	16 (88.9)	143 (94.7)	211 (92.5)	0.54
Non-STEMI	3 (16.7)	12 (7.9)	20 (8.8)	0.46
CVA	0 (0)	2 (1.3)	6 (2.6)	0.64
Renal failure	1 (5.6)	1 (0.8)	0	0.03
Drug history				
Aspirin	15 (83.3)	132 (87.4)	164 (71.9)	0.001
ACE inhibitor	11 (61.1)	98 (64.9)	126 (55.2)	0.16
ARB	1 (5.6)	9 (6.0)	6 (2.6)	0.25
Beta-blocker	13 (72.2)	120 (79.4)	153 (67.1)	0.02
Nitrate	12 (66.7)	122 (80.7)	150 (65.7)	0.004
Calcium channel blocker	0 (0)	8 (5.2)	14 (6.1)	0.53
Plavix	12 (66.7)	105 (69.5)	121 (53.0)	0.004
Lipid lowering agent	7 (38.9)	57 (37.7)	56 (24.5)	0.01
Statin	6 (33.3)	58 (38.4)	76 (33.3)	0.59
Glucose lowering agent	6 (33.3)	21 (13.9)	36 (15.7)	0.11
Insulin	2 (11.1)	4 (2.7)	5 (2.1)	0.13
Warfarin	1 (5.6)	1 (0.7)	1 (0.5)	0.18
Verapamil	0 (0)	5 (3.3)	7 (3.0)	1
Adenosine	4 (22.2)	43 (28.4)	18 (7.8)	<0.001
Integrilin	10 (55.5)	86 (56.9)	60 (26.3)	<0.001
SBP	135.0 (119.0, 155.0)	131.0 (120.0, 150.5)	130.0 (120.0, 150.0)	0.99
DBP	77.0 (70.0, 80.0)	85.0 (70.0, 100.0)	81.0 (75.0, 97.5)	0.09
Heart rate	72.0 (60.0, 95.5)	78.5 (68.7, 90.0)	78.0 (65.0, 86.0)	0.71
Hb	14.57 ± 1.57	14.61 ± 1.64	14.76 ± 1.79	0.71
Hematocrit	42.30 ± 3.68	43.04 ± 4.06	43.44 ± 5.44	0.82
WBC	11054.55 ± 3488.94	10261.22 ± 3178.09	10610.40 ± 3681.88	0.56
Platelets	212000 (167550, 261000)	215000 (174000, 264000)	223000 (189750, 257250)	0.51
MPV	10.43 ± 0.85	9.77 ± 1.06	9.85 ± 0.90	0.18
FBS	169.0 (100.0, 228.0)	119.0 (102.0, 158.0)	116.0 (97.0, 156.7)	
BS	186.0 (138.7, 348.0)	144.0 (113.0, 199.0)	141.0 (114.5, 205.0)	0.18
HbA1c	9.54 ± 1.43	8.36 ± 2.46	8.45 ± 2.04	0.54
Total Cholesterol	176.80 ± 47.93	184.00 ± 46.10	183.39 ± 41.02	0.82
Triglyceride	139.0 (83.0, 177.0)	129.0 (90.0, 176.0)	138.0 (99.0, 178.0)	0.56
LDL	106.13 ± 37.97	114.55 ± 38.85	113.90 ± 35.27	0.7
HDL	42.0 (26.0, 53.0)	41.0 (35.0, 47.0)	41.0 (36.0, 47.0)	0.91
Creatinine	1.1 (1.0, 1.2)	1.0 (0.8, 1.1)	1.1 (0.9, 1.2)	0.004
Global EF	50.0 (35.0, 50.0)	45.0 (37.75, 50.0)	45.0 (35.0, 55.0)	0.78
right ventricular diameter	3.25 (3.0, 4.0)	3.2 (2.9, 3.5)	3.0 (3.0, 3.5)	0.21
* P-value ≤ 0.05 was considered as statistically significant				

The comparison of the procedural parameters showed that the rate of angioplasty in the TIMI flow grade-3 subgroup was significantly less than the one in other groups while right coronary artery had a more frequency in this group. Post dilatation maximum pressure of balloon inflation is importantly raised in the TIMI-3 subgroup. On the other hand, thrombosis migration and ostial dilatation was significantly more observed at the TIMI of flow type 0-1 subgroup (P<0.001 for both variables). The long tubular lesion was significantly more observed at the flow of TIMI type 3 subgroup (P=0.004) while the diffuse lesion was further widespread in the TIMI flow grade 0-1 subgroup (P=0.008). The total occlusion and dissection was more observed in the TIMI flow grade 0-1 subgroup (P=0.03). The details of the procedural variables are depicted and summarized in **Table 2**.

**Table 2:** Comparing the angiographic parameters between the TIMI subgroups

Parameter	TIMI = 0,1 (n=18)	TIMI = 2 (n=151)	TIMI = 3 (n=228)	P-value*
Reperfusion time	6.70 ± 3.75	8.16 ± 6.98	7.42 ± 7.52	0.22
Target vessel				0.001
LAD	13 (72.2)	110 (72.8)	125 (54.8)	
LCX	1 (5.7)	9 (5.9)	36 (15.8)	
RCA	3 (16.7)	32 (21.2)	66 (28.9)	
SVG	1 (5.6)	0.0	1 (0.4)	
AHA grade (B2, C)	14 (77.7)	127 (86.4)	193 (84.6)	0.88
Number of lesions	2.0 (2.0, 2.0)	2.0 (2.0, 2.0)	2.0 (2.0, 2.0)	0.4
length of Lesion	19.9 (15.2, 25.7)	21.5 (16.0, 28.5)	20.0 (15.0, 27.5)	0.07
Stent diameter	3.5 (2.7, 3.5)	3.0 (3.0, 3.5)	3.0 (2.7, 3.5)	0.18
Stent length	18.0 (12.0, 24.0)	23.0 (18.0, 28.0)	20.0 (18.0, 28.0)	0.09
Stent inflation pressure	12.0 [(9.0, 14.0)	12.0 (12.0, 14.0)	12.0 (11.0, 14.0)	0.46
Post dilatation Maximum balloon inflation pressure	11.0 (7.0, 12.0)	16.0 (14.0, 19.5)	18.0 (14.0, 20.0)	0.008
Maximal inflation pressure	10.0 (8.0, 11.0)	10.0 (8.0, 12.0)	10.0 (8.0, 12.0)	0.72
New thrombectomy	4 (22.2)	31 (21.2)	21 (9.7)	0.005
Persistent dye stasis distal to occlusion	4 (22.2)	19 (11.1)	16 (6.1)	0.04
Thrombosis migration	8 (44.4)	23 (15.2)	13 (5.7)	<0.001
Ostial lesion	4 (22.2)	7 (4.6)	5 (2.2)	<0.001
Proximal lesion	6 (33.3)	78 (51.7)	100 (43.9)	0.17
Non-proximal	8 (44.4)	62 (41.1)	111 (48.7)	0.34
Long tubular	4 (22.2)	41 (27.2)	97 (42.5)	0.004
Diffuse lesion	13 (72.2)	94 (62.3)	110 (48.2)	0.008
Calcified lesion	0 (0)	4 (2.6)	13 (5.7)	0.23
Bifurcation	0 (0)	12 (7.9)	8 (3.5)	0.09
Eccentric	0 (0)	18 (11.9)	49 (21.5)	0.008
Tortuous or angulated lesion	7 (38.9)	68 (45.4)	78 (34.2)	0.1
Proximal segment tortuosity				0.77
Mild	11 (61.1)	101 (98.1)	124 (96.9)	
Severe	0 (0)	2 (1.9)	4 (3.1)	
Angulated segment	0 (0)	7 (6.7)	10 (7.8)	0.62
Thrombus	7 (38.9)	46 (30.5)	54 (23.7)	0.17
Total occlusion	15 (83.3)	107 (70.9)	139 (61.0)	0.03
Degenerated vein graft	1 (5.6)	0 (0)	1 (0.4)	0.08
Procedure				0.01
Direct stenting	1 (5.6)	21 (13.9)	28 (12.3)	
Primary stenting	11 (61.1)	108 (71.5)	173 (75.9)	
Secondary stenting	3 (16.7)	6 (4.0)	18 (7.9)	
Ballooning	3 (16.7)	16 (10.6)	9 (3.9)	
Stent type				0.69
Bare metal stent	14 (77.7)	110 (81.5)	178 (81.3)	
First generation drug eluting stent	1 (5.6)	11 (8.1)	22 (10.0)	
Second generation drug eluting stent	0	14 (10.4)	19 (8.7)	
Side branch occlusion	0 (0)	2 (3.0)	0	0.12
Dissection	1 (5.6)	0	5 (2.2)	0.06
Result				0.001
Successful	15 (83.3)	145 (96.0)	227 (99.5)	
Acceptable	3 (16.7)	6 (4.0)	1 (0.4)	
Type of PCI				0.9
Single vessel	15 (83.3)	130 (86.1)	198 (86.8)	
Multivessel	3 (16.7)	21 (13.9)	30 (13.2)	
Territory				0.61
Single territory	16 (88.9)	139 (92.1)	203 (89.0)	
Multi territory	2 (11.1)	12 (7.9)	25 (11.0)	
LAD territory	12 (66.7)	113 (74.8)	135 (59.2)	0.007
RCA territory	4 (22.2)	36 (23.8)	78 (34.2)	0.07
LCX territory	18 (100)	151	227	
* P-value ≤ 0.05 was considered as statistically significant				
AHA = American Heart Association,				

In the regression analysis, the predictors for TIMI flow grade were utilize of adenosine or Integrilin, history of diabetes mellitus, POIT, long tubular lesion, and lesion in the LAD territory. Prophesier of TIMI flow type are showd in **Tab. 3**.

**Table 3:** Independent predictors of TIMI flow score

Predictor	Odds ratio	CI 95%	P-value*
Adenosine	2.45	1.32-4.52	0.004
Integrilin	3.05	1.89-4.90	<0.001
Diabetes mellitus	1.73	1.04-2.85	0.03
POIT	0.37	0.22-0.63	<0.001
Long tubular lesion	0.55	0.33-0.91	0.02
LAD territory	2.07	1.25-3.46	0.004

**Fig. 1:**
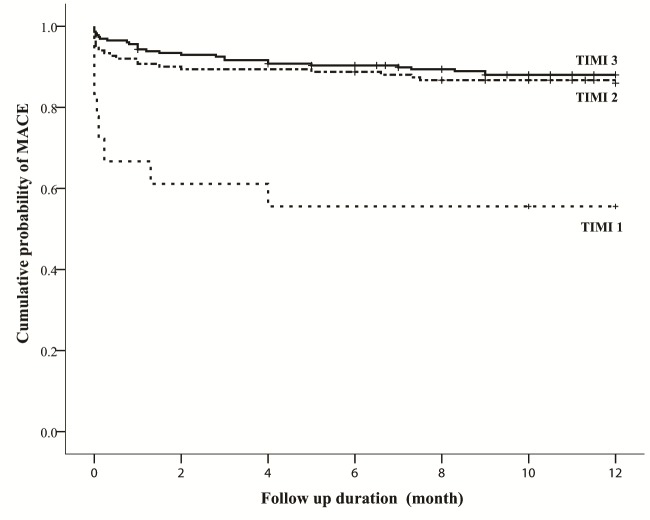
The survival of the patients with a TIMI

The occurrence of the 12-months MACE at this research people was 14.1%. But, the impact of MACE is importantly larger in patients with a flow of TIMI type of 0-1 (P=0.001). That is not non-cardiac mortality in any of the subgroups but the frequency of cardiac mortality is importantly larger in the flow of TIMI type of 0-1 subgroup (P<0.001). The frequency of MACE within each TIMI flow grade subgroup and the comparison between these subgroups are listed in **Table 4**. Similarly, the survival of the patients with a TIMI flow grade of 0-1 were significantly poorer than the ones of the other two subgroups (Hazard ratio =4.96; 95% confidence period: 2.25-10.94; P<0.001) (**Fig. 1**). 

**Table 4:** Comparing the frequency of the 12-months MACE between the TIMI subgroups

Parameter	TIMI = 0-1 (n=18)	TIMI = 2 (n=151)	TIMI = 3 (n=228)	P-value*
In-hospital mortality	4 (21.1)	10 (6.6)	5 (2.2)	0.007
TVR	0 (0)	7 (4.6)	16 (7.0)	0.34
TLR	0 (0)	2 (1.3)	5 (2.2)	0.69
Non-fatal MI	1 (5.6)	5 (3.3)	8 (3.5)	0.88
CABG	0 (0)	5 (3.3)	10 (4.4)	0.59
Cardiac death	8 (44.4)	11 (7.3)	7 (3.1)	<0.001
Mortality	8 (44.4)	11 (7.3)	7 (3.1)	<0.001
Total MACE	8 (44.4)	21 (13.9)	27 (11.8)	0.001
CABG = Coronary artery bypass graft,				

## Discussion

In the research, that rate of slow/ no-reflow was 4.5%, which is comparable to previous reports [**19**-**20**]. It was observed that the flow of TIMI type was affected with the utilizing of adenosine or Integrilin, history of diabetes mellitus, POIT, long tubular lesion, and lesion in the LAD territory. Moreover, patients with a low TIMI grade have a high risk for MACE and thereby a poor survival. 

The no-reflow event, or coronary marked impairment flow without evident obstruction or embolization of distal, is seen in about 2%-11% of all coronary procedures, depending of the indication and type of intervention [**21**]. Therefore, the flow of TIMI type was a useful tool for the through the measure of coronary blood flow and the lamination of the cases after the procedure.

Several different predictors for slow/ no-reflow phenomenon, like a the C-reactive protein, atrial natriuretic peptide [**22**], endothelin-1 [**23**], thromboxane A2 [**24**], intraplatelet melatonin [**25**], white blood cellule number [**26**], or plasma glucose level at admission [**27**], and composition of the culprit plaques in intracoronary ultrasound [**28**,**29**], should done recognized in previous studies. It must be marked that not all of these factors can be evaluated in routine practice and more applicable factors were required to be implemented. Therefore, in the current research, the factors of clinical that were routinely used in the management of PCI patients were evaluated and it was found out that the use of adenosine or Integrilin, history of diabetes mellitus, POIT, long tubular lesion, and lesion in the LAD territory were the independent predictors for slow/ no-reflow. Although diabetes mellitus was identified via a prophesier for no-flow [**30**], old age was not a predictor in our study despite the previous report [**31**]. 

Clinical factors that can be assessed before the procedure are more important as they provide a prospect for the clinicians to consider all the necessary measures for preventing slow/ no-reflow during the procedure beforehand. These measures can include the check of blood glucose in diabetic cases [**32**] or the utilize of glycoprotein IIb/ IIIa inhibitors [**33**,**34**]. 

In the current research, TIMI flow is importantly connected by MACE. This was consistent with previous studies that suggested the TIMI flow grade via a prophesier for MACE [**35**,**36**]. Moreover, PCI have been shown as an effective method in the treatment of patient with chronic total occlusion [**37**]. Hence, it seems that the final TIMI flow grade is the main predictor for MACE rather than the initial coronary flow state. Considering the literature and our findings, the final TIMI flow grade can be suggested as a useful predictor for the survival in AMI patients who undergo primary PCI. We also presumed that patients with persistent no-flow might be proper candidates for pharmacomechanical treatment strategies, such as the therapy by glycoprotein IIb/ IIIa inhibitors.

**Study limitations**

There are some limitations to our study. First, this was a single-center, retrospective study. Secondly, glycoprotein IIb/ IIIa inhibitors were used in limited cases due to their price in Iran and our current guidelines. The follow up period of the cases was of 12 months and longer durations may help in better evaluating the prophesier of the TIMI flow. Diabetes mellitus was diagnosed based on the patient’s history or the use of glucose lowering agents and we didn't decide the patients for glucose intolerance. 

## Conclusion

Briefly, the utilize of adenosine or Integrilin, history of diabetes mellitus, POIT, long tubular lesion, and lesion in the LAD territory can be used as predictors for the lower TIMI grade. Moreover, cases by a lower TIMI grade have a higher MACE and a lower survival.

**Acknowledgments**

This research is backed by the Heart Center of Tehran and Medical Sciences Tehran University

**Interest Conflict**

The writers have no interest conflict to declare.
